# Neural correlates of consciousness

**DOI:** 10.1111/nyas.12257

**Published:** 2014-08-28

**Authors:** Geraint Rees

**Affiliations:** UCL Institute of Cognitive Neuroscience and Wellcome Trust Centre for Neuroimaging, University College LondonLondon, United Kingdom

**Keywords:** fMRI, vision, consciousness, awareness, neglect, extinction

## Abstract

Jon Driver's scientific work was characterized by an innovative combination of new methods for studying mental processes in the human brain in an integrative manner. In our collaborative work, he applied this approach to the study of attention and awareness, and their relationship to neural activity in the human brain. Here I review Jon's scientific work that relates to the neural basis of human consciousness, relating our collaborative work to a broader scientific context. I seek to show how his insights led to a deeper understanding of the causal connections between distant brain structures that are now believed to characterize the neural underpinnings of human consciousness.

## Introduction

Our awareness of the external world is central to our everyday lives. People consistently and universally use verbal and nonverbal reports to indicate that they have subjective experiences that reflect the sensory properties of objects in the world around them. This awareness of the external environment is contingent on sensory processing, and can be disrupted by damage both to sensory cortices and to other areas of the human brain. In our work together, Jon and I explored some of the ways in which the causal interplay between different brain regions affected sensory processing, and that in turn affected our reports of objects in the external environment. In doing so, he provided some key insights into the neural basis of consciousness and how it can be particularly affected by damage to the parietal cortex.

A common thread throughout Jon's research, and one that is readily apparent throughout this special volume, is an interest in how sensory processing is modulated by top-down influences. While basic sensory processes have been well characterized by many investigations using psychophysics and in sensory physiology, Jon's work has significantly increased our understanding of the ubiquity of higher-level influences on sensory processing. Techniques for noninvasive measurement of human brain activity such as functional magnetic resonance imaging (fMRI), positron emission tomography, electroencephalography (EEG), and magnetoencephalography (MEG) can reveal the neural substrates of sensory processing in the human brain, and together we used these approaches to explore the neural basis of awareness.

## Sensory processing and visual awareness

Vision is a common model system in which to approach the question of whether certain neural processes are specifically correlated with awareness of objects in the external world. This is because vision plays a central role among sensory modalities, and a comparatively large amount is already known about the anatomy and physiology of the mammalian and primate visual systems that can be used to interpret responses in the human brain. The principal methodological approach employed is to contrast the neural activity evoked by conscious versus unconscious information processing.^1^ This contrast permits studies investigating what is special about the neural activity specifically associated with the contents of conscious experience. A large body of work has now established that changes in the contents of consciousness without corresponding changes in physical stimulation are reliably associated with changes in activity in the ventral visual cortex, in areas known to represent the attributes represented in the contents of consciousness.^2^,^3^ Thus, activity in the visual pathway can reflect conscious processing of visual stimuli. However, stimuli that are not consciously perceived can also evoke some activation of the same cortical areas, sometimes with comparable amplitude.

Such observations are illustrated by a study^4^ we undertook to identify how brain activity reflected the visibility of a very simple visual stimulus. We used metacontrast masking, a type of backward masking where a briefly presented simple visual target stimulus is rapidly followed by a nonoverlapping mask that shares a contour with the target. In such a situation, observers often cannot report the presence of the target, and changes in the interval between target and mask presentation cause systematic changes in the proportion of trials in which the target is accurately reported. This therefore allows us to parametrically manipulate the visibility of a simple visual stimulus. In these circumstances, we showed that signals measured using fMRI from stimulus-driven areas of early visual cortex did not reflect parametric changes in the visibility of the stimulus. The psychometric visibility function was instead correlated with activity in later visual regions plus the frontal and parietal cortex, and surprisingly in representations of the unstimulated stimulus surround for the primary visual cortex. However, a critical observation from this study was that decreased stimulus visibility was associated with a regionally specific decoupling between the early visual cortex and higher visual areas. Thus, not only does activity in stimulus-driven areas of the early visual cortex not necessarily reflect awareness, but also dynamic changes in coupling between the early visual cortices and higher visual areas can closely reflect visual perception.

**Figure 1:**
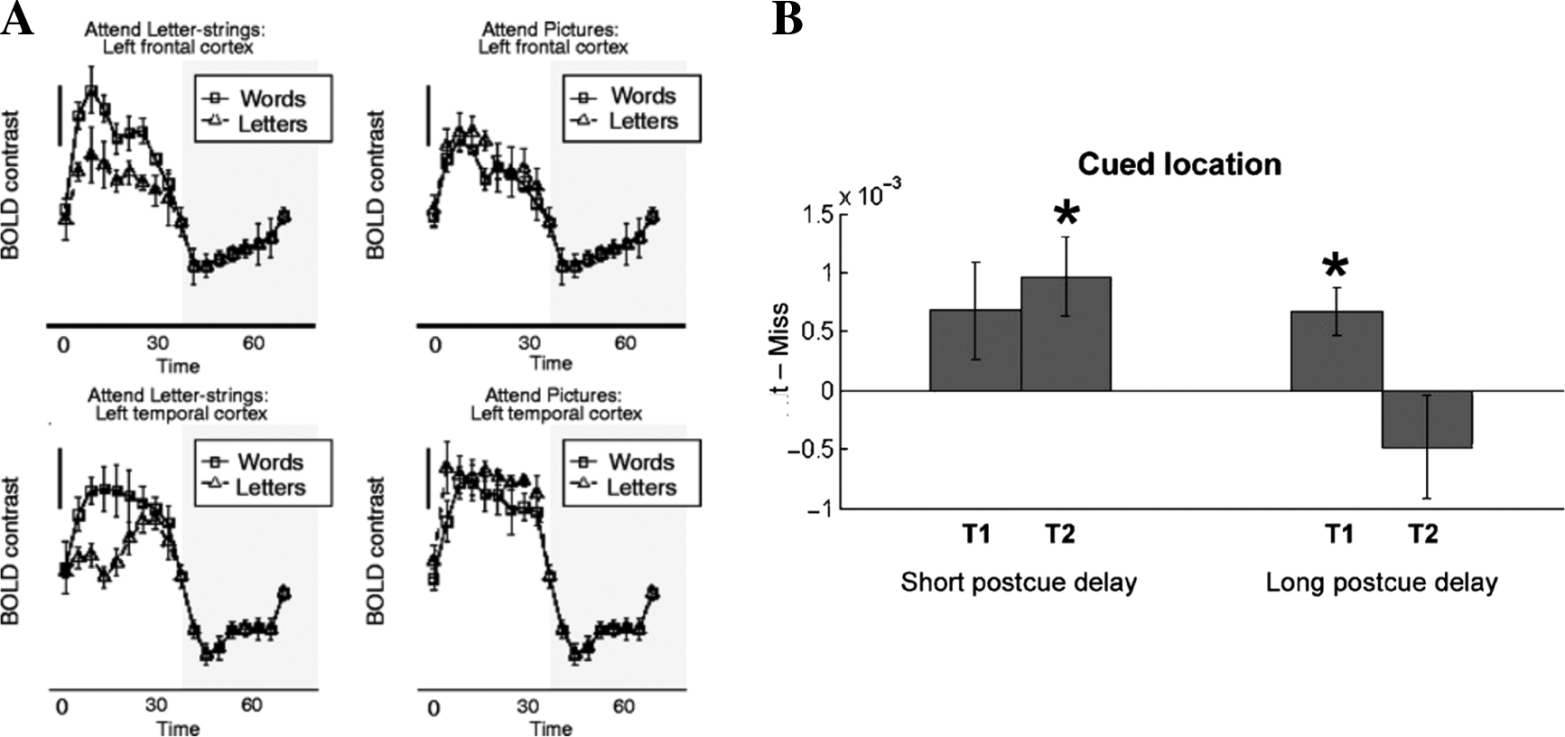
(A) Activity in the ventral visual and prefrontal cortices associated with processing of fixated words is modulated by attention. Shown in each of four panels is the average BOLD signal measured using functional MRI in a group of participants attending to streams of superimposed letters and pictures. Error bars indicate inter-participant standard error, and the dark scale bar represents 0.5% BOLD signal change. Activity evoked when the letter stream contained meaningful words is plotted with black squares and a solid line, and activity when the same stream included only meaningless letter strings is plotted with triangles and a dotted line. Data taken from Ref. 6. (B) Top-down modulation of the human early visual cortex after stimulus offset associated with successful postcued report. Shown are averaged BOLD responses for hits (successful identification) versus misses at stimulus locations in the primary visual cortex that were cued by an auditory postcue, measured before (*T*_1_ = 0–4.16 s) or after (*T_2_* = 4.29–8.45 s) the auditory cue began to influence the hemodynamic response. A significant difference between hits and misses was observed only in the later time window (*T*_2_) when the postcue was presented at a short delay; when the postcue was presented at a long delay, a significant difference was only observed in the initial time window (*T*_1_). *P < 0.05. Data from Ref. 9.

If coupling between different areas of the sensory cortex can be important for visual awareness, then we need to understand how top-down signals of all kinds can influence sensory processing associated with awareness. The crucial role of top-down signals in determining whether information impinging on the senses reaches awareness is dramatically illustrated by the phenomenon of inattentional blindness.^5^ When an individual's attention and effort are fully engaged in a demanding task, even clearly presented objects can fail to be noticed, particularly when unexpected. It has been unclear whether this failure to report the content of ignored information reflects a complete failure to perceive it (inattentional blindness) or merely that it is processed and perceived but rapidly forgotten (inattentional amnesia). Jon and I used fMRI to shed light on this question by comparing the brain activity elicited by words (compared to nonword letter strings) presented in a rapid stream at fixation together with superimposed line drawings.^6^ When attention was directed to the words, compared to letter strings, activity was evoked in a number of different brain regions, including regions of the ventral visual cortex associated with processing single words. But when attention was fully engaged in attending to the overlapping line drawings, brain activity no longer differentiated between words and meaningless letter strings, even though they were presented directly at fixation (Fig. 1). These results demonstrate inattentional blindness in the direct sense of the word, and showed that visual recognition depends wholly on attention, even for highly familiar and meaningful stimuli at the center of gaze. While awareness of the words was only assessed indirectly in this study, through a subsequent memory test, this demonstrates how signals in the visual cortex are dramatically modulated by task performance (here, a very attentionally demanding task).

The modulation of sensory processing in early visual areas by top-down signals therefore plays an important role in conscious perception. But this study, like many others addressing similar issues,^7^ has focused on the effects of top-down signals either immediately before (e.g., an attentional cue) or (as here) during visual presentation. More recently, important theories of visual awareness have suggested that sensory processing and conscious visual perception may also depend on late top-down influences from recurrent or feedback processing. These effects may potentially come about after a visual display, when different modulatory effects can be seen. For example, we showed that reward signals have retinotopically specific effects on visual cortices after stimulus processing.^8^ But to test for a more direct link between such top-down signals after a stimulus has been presented on visual awareness, we employed a postcue report procedure to manipulate the visibility of stimuli.^9^ This exploited the observation that symbolic postcues presented in a different modality (e.g., auditory) after a visual display can nevertheless influence the ability to see (and thus report) the presence or identity of a target at a specific spatial location in that previously presented display. By measuring brain activity using fMRI during such a procedure, we confirmed such behavioral findings but showed that they were associated with enhanced target-specific signals in the human early visual cortex (both primary visual cortex and V2). Auditory postcues presented very shortly after the target display (200 ms) were effective in facilitating correct conscious report, and the strength of the corresponding modulation of target-associated signals in the visual cortex predicted individual differences in behavioral performance. In contrast, auditory postcues presented at a slightly later (1000 ms) time failed to facilitate correct conscious report, although they still had some target-specific effects on activity in the early visual cortex (Fig. 1). These findings show that, consistent with previous behavioral reports, there is a critical and brief window after a visual stimulus has disappeared in which an appropriate postcue can lead to successful report of an otherwise unseen stimulus. Moreover, successful conscious report of such a postcued stimulus is related to the strength of top-down modulation provided by the postcue in the early visual cortex. This suggests a framework in which sensory representations of visual stimuli are flexibly influenced by top-down modulatory signals both before and after a stimulus is presented, and in which the interplay of such signals jointly leads to visual awareness.

## Awareness in other sensory modalities

Although most of our work together focused on the human visual system, all of the senses contribute to awareness of the external world, and so it is an open question whether similar principles apply to how awareness relates to sensory processing in other modalities. Different sensory modalities such as hearing, touch, and vision share a common principle of cortical organization, in that elementary features of the sensation are topographically mapped across the cortical surface. The auditory cortex contains a tonotopic map; the visual cortex is retinotopically mapped; and the somatosensory cortex is mapped according to a topographic representation of the bodily surface. In the visual cortex, when a stimulus is consciously perceived at a particular location in the visual field, the corresponding location in the retinotopic cortex is activated even when there is no direct physical stimulation at that visual field location. For example, activity in the retinotopic cortex can be seen on the path of apparent motion where no stimulus is physically present, and this reflects top-down signals from higher visual areas involved in the processing of visual motion.^10^ Jon and I discovered a similar principle operates in the human somatosensory cortex.

We investigated the neural correlates of a robust somatosensory illusion where a stimulation, occurring initially at the wrist, then twice in rapid succession further up the arm, can create the illusion of sequential touches at intervening locations along the arm that were never stimulated. This dissociation of tactile perception from physical stimulation is sometimes known as the *cutaneous rabbit* illusion, in which one experiences a sensation as if a tiny rabbit is hopping along the arm from wrist to elbow. We exploited the established somatotopy of the primary sensory cortex in humans and used fMRI to examine signals in the sensory cortex corresponding to the location of the wrist, elbow, or intervening forearm while people were stimulated in a fashion that induced the illusion.^11^ We compared signals when the illusion was elicited with those produced by control stimulation at the same sites on the wrist and elbow, but now in a different order that did not induce the cutaneous rabbit illusion. We found that compared to control stimulation, illusory perception of the cutaneous rabbit activated the contralateral primary somatosensory cortex at a location precisely corresponding to the somatotopic location of the filled-in illusory percept on the forearm. The amplitude of this activation, even though no stimulus was physically present at that location, was comparable to that elicited by veridical stimulation at the intervening position on the forearm. Thus, illusory somatosensory percepts lead to modulation of signals in the primary somatosensory cortex in a somatotopic fashion directly analogous to similar illusory percepts in the visual domain. Moreover, the cutaneous rabbit illusion also activated areas of the premotor and prefrontal cortices. As with conscious visual perception, conscious somatosensory perception is associated not only with modulation of activity in the primary sensory cortices but also with areas outside sensory processing areas in the prefrontal cortex.

## Visual awareness after damage to the parietal cortex

Jon's work, together with that of others,^12^ thus shows that perceptual awareness is not determined by the stimuli impinging on our senses, but by which stimuli we choose to attend to. This key insight into normal cognition helps us understand the nature of spatial neglect, another of Jon's enduring interests.^13^,^14^ Unilateral spatial neglect is a common and disabling neurological disorder after unilateral brain damage, particularly to the parietal cortex. It is characterized by a lack of awareness of sensory stimulation occurring on the contralesional side of space, combined with a deficit in orienting and exploratory behaviors that would normally be directed toward that side. But although neglect apparently involves a dramatic loss of awareness of sensory events occurring on the affected side, paradoxically such a loss can occur even though the primary sensory pathways for processing the neglected stimuli may still all be intact. Accordingly, although patients may show profound neglect for multisensory stimuli, not only are their sensory pathways for processing these stimuli intact, but also under appropriate conditions they can sometimes report awareness for isolated stimuli. Such a paradox can be resolved by understanding neglect as a pathological restriction in the normal ability to choose which stimuli to attend to.

Unilateral spatial neglect can be observed in different forms after a wide variety of unilateral brain lesions, but is most common and long lasting when damage involves the inferior parietal lobe in the right hemisphere. The effect of neglect on patients’ ability to report visual experience contralesionally demonstrates that signals from outside the visual cortex are important in determining whether sensory information reaches awareness.^13^,^14^

Visual extinction is often associated with visual neglect, a sign classically associated with right parietal damage. Patients with visual extinction can see a single stimulus presented in the ipsilesional or contralesional visual field, but are characteristically unaware of the same contralesional stimulus during simultaneous stimulation of both fields. The ipsilesional stimulus is said to extinguish the contralesional stimulus from awareness during bilateral stimulation, perhaps due to a pathological bias in attention toward the ipsilesional side. Psychophysical evidence suggests that, although extinguished stimuli are not consciously seen, they may undergo residual processing and exert implicit effects on performance.^13^ We therefore set out to use noninvasive imaging to delineate the neural structures mediating such residual processing for extinguished stimuli.

**Figure 2:**
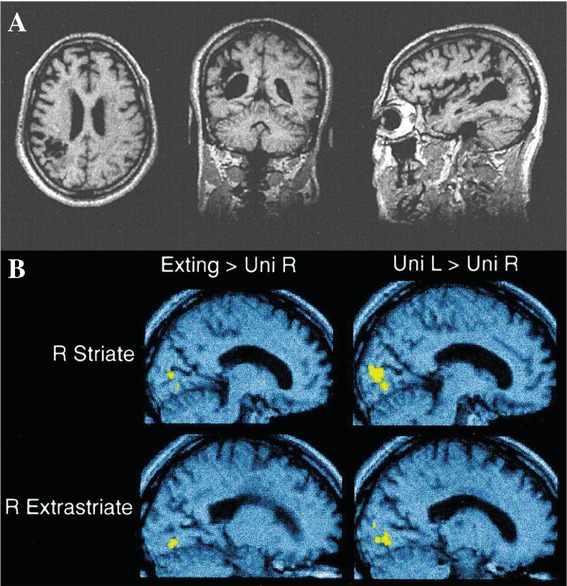
(A) Anatomic location of the lesion causing neglect and extinction in the patient studied in Refs. 15–17. Three sections (axial, coronal, and sagittal) are shown through a T_1_-weighted MRI. A low attenuation area within the right inferior parietal lobule, consistent with an old cerebral infarction, is shown. Note the highly circumscribed nature of the lesion, sparing visual cortex structurally. (B) From Ref. 15, cortical areas activated by consciously seen unilateral stimuli in the left visual field and by extinguished left visual field stimuli on bilateral trials. These panels show the location of the largest and most reliable activations, revealed either by the comparison of bilateral trials (showing extinction) minus unilateral right trials (left panels), or by the comparison of unilateral left trials minus unilateral right trials (right panels). The *x*-coordinate for the slice shown was determined by the peak activation of the comparison it depicts. The upper panels show loci of activation in the striate cortex; the lower panels show those in the early extrastriate area. All activations are superimposed on the T_1_-weighted anatomical image from panel A. The similar location of activations for the two contrasts is evident.

We used event-related fMRI to study the neural activity evoked by an extinguished visual stimulus in a patient with very focal right inferior parietal damage and profound left-sided extinction.^15^ Different images of either faces or houses were presented in the left or right field, either unilaterally or bilaterally on each trial. The patient was required to indicate by button press whether he saw an object on the left, the right, or on both sides. He usually saw only the right object on bilateral trials, yet our fMRI analyses revealed activation of the visual cortex contralateral to the extinguished left stimulus on these trials (compared with right-only stimulation), in both the primary visual cortex and the ventral visual areas of the right hemisphere (Fig. 2). Critically, the patient's brain activity had a similar location and time course to that resulting from a single stimulus (which was clearly seen by the patient) in the left versus the right visual field. This work thus established that the cortical pathways involved in the normal processing of a single seen stimulus can thus still be activated by an unseen, extinguished stimulus after right parietal damage.

We also investigated the stimulus specificity of brain areas activated by stimuli in the left visual field of the same patient.^16^ Critically, we found that an extinguished face stimulus presented in the left visual field produced strong category-specific activation of the right fusiform face area. Moreover, on trials in which a face presented in the left visual field was seen consciously (rather than extinguished), there was more activity in both the ventral visual cortex and in the frontal and parietal areas of the undamaged left hemisphere. Thus, not only are cortical pathways involved in the normal processing of a single seen stimulus also activated by an unseen extinguished stimulus, but also higher levels of the ventral visual pathway retain their category specificity after parietal damage.

In further work with other colleagues, Jon subsequently studied how the emotional valence of visual stimuli affected residual processing in neglect and extinction.^17^ He used a similar paradigm, but with faces that had either neutral or fearful expressions. As with the previous work, seen faces in the left visual field activated the primary visual cortex in the damaged right hemisphere and other areas further along the ventral visual pathway. Faces that were extinguished and unseen also activated the early visual cortex and the ventral visual pathway. But crucially, the left amygdala (as well as areas in the ventral visual pathway) showed differential activation to fearful (versus neutral) faces whether they were seen or extinguished. Indeed, amygdala activation did not distinguish between perceived and unseen faces. Thus unconscious residual processing is not restricted to cortical structures but can also be clearly identified in subcortical pathways associated with emotional processing after parietal damage leading to visual extinction.

Taken together, these findings from fMRI studies converge well with event-related potential (ERP) data (see Ref. 18 for a review) to show extensive residual sensory processing of extinguished visual stimuli that escape awareness. By providing such evidence, they identify preserved function upon which efforts at rehabilitation can potentially build.

Jon was interested not only in visual processing, but also on the effects that cross-modal stimulation had on awareness after parietal damage. With other authors, he studied the same patient with neglect and extinction following focal parietal damage, but now investigating interactions between touch and vision.^19^ The patient exhibited cross-modal extinction, such that a touch on the left index finger could be extinguished from awareness by concurrent stimulation of the right visual field. Nevertheless, even when such extinction of touch occurred, fMRI revealed consistent activation of the contralateral primary somatosensory cortex, suggesting preserved unconscious residual processing. Similar to vision, awareness of touch led to additional activation in the frontal and parietal cortices; and on trials where cross-modal extinction arose, preserved right parietal cortex (i.e., unaffected by the lesion) was coupled more strongly with the left visual and right somatosensory cortex that was activated by the competing visual and tactile stimuli. This suggests that cross-modal extinction is a consequence of competitive interactions between sensory representations that are mediated by the multimodal (here, parietal) cortex. More generally, these findings show that the general principles of unconscious residual processing for extinguished stimuli extend beyond visual processing to apply to touch as well.

## Using causal interventions to study visual awareness

A common theme running through Jon's work, both in relation to awareness and more generally with respect to his broader interests in cognition, was an abiding interest in the nature of the causal interplay between brain regions. Recently, Jon was building upon the work reviewed here and elsewhere^a^ to develop exciting new methods for direct, noninvasive study of causal interactions. Work on the neural correlates of consciousness has often focused on the role of particular brain regions, and sometimes under an implicit assumption that each is operating in isolation. But as Jon's work shows, each area is embedded in a much wider network of interacting brain areas within remote but interconnected regions of the brain. To directly investigate these phenomena, Jon developed the use of altering activity in a focal brain area using transcranial magnetic stimulation (TMS) while measuring the effect of such an intervention on remote but interconnected brain regions using concurrent fMRI. The concurrent use of TMS and fMRI is technically challenging, but Jon's early work was already demonstrating how this could potentially be used to make powerful inferences about visual perception and ultimately about visual awareness.

For example, we examined the effects of applying TMS to the human frontal eye field (FEF) on visual representations in the retinotopic visual cortex.^20^ Strikingly, FEF TMS led to increases in activity in retinotopic regions that represented the peripheral visual field but decreases in activity in those regions of the visual cortex representing the central visual field. These top-down causal influences were independent of whether the visual cortex was processing visual information. Moreover, these top-down signals had a differential effect on visual awareness, because TMS applied to the FEF enhanced perceived contrast for peripherally presented stimuli compared to centrally presented stimuli, in accordance with the differential effect on retinotopic signals. This study provided causal evidence that circuits associated with the human FEF can modulate activity in the human retinotopic visual cortex in a topographically precise fashion that has consequences for conscious visual perception. Jon further developed this technique to investigate how such top-down causal influences also depended on task, and it is now readily apparent how future work could apply this powerful new technique to investigate the causal nature of the functional interactions between remote but interconnected brain regions that characterize the neural correlates of human consciousness.

## Conclusions

Jon's work shed important new light on normal brain function relating to our conscious perception of the world, and on disabling cognitive deficits after brain injury that impaired such perception. It is now orthodoxy to state that visual awareness depends on interplay between activities in the cortices specialized for visual processing and the parietal and prefrontal cortex as part of a distributed network. Jon's work played a key role in establishing such orthodoxy through compellingly showing that study of higher-level modulatory influences provides a tractable handle on causal interplay between different psychological systems, and at a mechanistic level between different brain regions.

## Footnotes

a Miller, M.B. and A. Kingstone, eds. 2013. The Year in Cognitive Neuroscience. Series of, *Ann. N.Y. Acad. Sci*. **1296:** 1–157.
